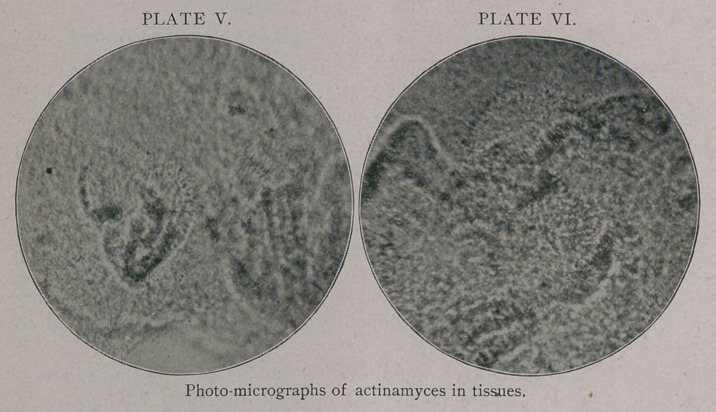# Actinomycosis Bovis, or “Lump Jaw”*Electrotypes kindly loaned by N. S. Mayo, D. V. S., Medical Society Experiment Station, Kansas State Agricultural College.

**Published:** 1893-03

**Authors:** R. R. Dinwiddie


### ACTINOMYCOSIS BOVIS, OR "LUMP JAW."*

By R. R. Dinwiddie, V. S.

This disease of cattle is one quite often observed in all countries where cattle are raised. While it occurs principally in cattle, cases of this disease have been reported in man, horses, dogs and pigs; but the occurrence of this disease in other animals than cattle is very rare.

This disease is commonly called "lump jaw," "big jaw," "big head," or "swelled head," and the lumps or tumors produced are known as "wens," "clyers," "cancers," "bone cancers," etc. When the tongue is the seat of the disease, this organ becomes hard and tense, and its usefulness is much impaired. This condition is commonly known as "wooden tongue."

This disease has been described by veterinarians and others as a cancer or tumor, under the following names, "fibroma," "myoma" and "osteosarcoma," until within the past fiteen years; since the true nature of the disease has been recognized, it has been described as *Actinomycosis bovis*.

This disease has attracted comparatively little attention until recently, and then principally on account of the peculiar pathological changes produced. The loss from the disease was very small. Animals were treated in the early stages of the disease and recovered, or they were sent to the shambles before they became seriously affected. Within the past five years, however, live-stock

* Electrotypes kindly loaned by N. S. Mayo, D. V. S., Medical Society Experiment Station, Kansas State Agricultural College.

sanitary commissions in some States have condemned animals affected with this disease, as suffering from a "dangerously contagious disease," and the flesh as "dangerous as food," and at the present time between two and three thousand cattle, annually, affected with this disease, are condemned and slaughtered at Chicago, St. Louis and other market places. The loss to stockmen, and the litigation resulting therefrom, have served to bring this disease prominently before the public.

### PREVALENCE OF THE DISEASE.

It is difficult to form a correct estimate of the prevalence of this disease, as it is quite generally distributed throughout the country.

In 1889, at the Union Stock Yards, Chicago, there were condemned and slaughtered 830 cattle affected with actinomycosis out of a total received of 3,023,281. This gives about one case of actinomycosis to every 3,642 cattle. In 1890 1,751 cases of actinomycosis were condemned out of a total of 3,484,280 cattle, which gives one case of actinomycosis to 1,990 cattle. In 1891, during 10 months, from January to November, 1,655 cases were condemned, and in 12 months, from November 1, 1891, to November 1, 1892, 1,888 cases were condemned. As I have not the total receipts of cattle at the yards for that time, I cannot form an estimate of the proportion. It will probably be in the neighborhood of one case of actinomycosis in every 1,600 or 1,700 cattle.

The great discrepancy in the number of cattle condemned in the years 1889 and 1890 must not be attributed to the rapid increase of the disease, but rather to the vigilance of the inspectors. Still, any estimate based upon the proportionate number of cases found in the Union Stock Yards will probably be much too low, as many stockmen do not ship the cattle affected with this disease, but dispose of them to local butchers. From my own observations I am of the opinion that one case of actinomycosis to 500 cattle will be a more correct estimate.

Observations *seem* to show that animals pastured upon low lands are more liable to contract this disease, also cattle fed upon rough feed, but this may be due to the greater danger of wounding the mucous membrane of the mouth, and thus affording a favorable opportunity for the organism to invade the tissues and cause the disease.

### SYMPTOMS OF THE DISEASE.

This disease is characterized by a lump or tumor, situated, usually, in the region of the head or throat. This tumor is caused by peculiar vegetable parasites which grow in the animal tissues; from their peculiar radiating, or star-shaped structure, they are called "actinomyces."

The first symptom of this disease is a slight swelling of the affected part, such as might result from an injury; in fact, many cases of actinomycosis appear to be caused by blows or injuries received by struggling in stanchions. The actinomyces must be present, however, in order that an animal may contract the disease. The enlargement gradually increases in size, and is usually well defined from the surrounding tissues. Upon manipulation, the tumor feels hard and dense, and, if not caused by the bulging of the adjacent bone, is usually attached to it. In the region of the throat it may be fluctuating. After a variable length of time the tumor softens in one or more places and discharges a rather thick, yellow and very sticky pus or matter. This discharge of pus may continue until the animal dies, or is disposed of. Uusually, however, the opening heals temporarily, only to go through the same process again. Often these tumors break and discharge the pus into the cavity of the mouth or throat. Sometimes, when a tumor breaks, a growth of new tissue protrudes from the opening, grows rapidly and resembles a cauliflower somewhat in appearance. Unlike an ordinary abscess, an actinomycotic tumor, after discharging pus, increases in size rapidly, until the tumor may reach the dimensions of a peck measure, or larger. In the later stages the teeth may become ulcerated and loosened and there is a drivelling of saliva from the mouth.

### AGE AT WHICH THE DISEASE OCCURS.

There seems to be no age when cattle are not subject to this disease, though I have never observed a case in a sucking calf. Most cases observed have been in two and three-year-old cattle. This may be accounted for partially by the fact that most cattle are "turned off" at this age, but as they are shedding their temporary molars, the irritated condition of jaws may offer favorable conditions for the reception and growth of the organisms which cause this disease.

There seems to be no especial time of the year when animals are more liable to contract this disease than another.

### LOCATION OF THE TUMOR

Of the location of the tumor I have found it to occur most frequently upon the lower jaw, next the upper jaw or face, throat and tongue, in the order named. Cases are reported where the disease has occurred in the lungs, liver, along the alimentary canal and in other parts of the body.

Plate I shows a case of actinomycosis of the lower jaw, one of the most common locations. In this case the tumor is caused by the bulging of the jaw-bone. The pus from this tumor was discharged into the mouth. The scars on the tumors show where in-

PLATE I.

*Actinomycosis bovis*, or lump jaw.

cisions were made to obtain material for inoculation and examination.

Plate II shows another common form, where the tumor is situated upon or within the bones of the face. This tumor discharged a little pus through the openings visible on the tumor, and which were made to obtain material. Most of the pus was discharged into the mouth.

COURSE OF THE DISEASE.

This disease is not rapidly fatal, and animals seldom die from the direct effects of the disease. The length of time an animal

PLATE II.

*Actinomycosis bovis*, or lump jaw.

may survive with this disease depends largely upon the location of the tumor and the rapidity of development. If the tumor is favorably situated, so it does not interfere seriously with the prehension or mastication of food, the animal usually survives several years. When death results from this disease, it is usually due to inanition the animal, being unable to gather or masticate its food properly, together with the drain upon the system by the discharge of pus, becomes emaciated and gradually dies of starvation. Several cases observed have suffered from this disease for five or six years, and would probably have survived several years more had they not been disposed of. Most cases of this disease are not allowed to run their course, the animals being treated in the early stages, or disposed of to local butchers or are destroyed.

### MORBID ANATOMY.

The lump or enlargement is the result, largely, of the multiplication of cells, principally of epitheloid and spindle-shaped connective-tissue cells. In this respect it differs from an ordinary abscess, where the enlargement is the result of an accumulation of pus As the growth of these cells in an actinomycotic tumor increases, they press against the surrounding tissues, producing the hard and dense condition of these tumors. On section through the tumor one of the first things noticed is the peculiar and rather disagreeable "nutty" odor which, I believe, is characteristic of this disease The outside of the tumor is a dense mass of fibrous connective tissue. Toward the center of the tumor the tissue is less dense and more vascular, being composed principally of epitheloid cells. In this tissue there are small, more or less globular, cavities containing a quantity of viscid pus. If this pus is spread out thinly upon a knife blade or bit of glass, and examined carefully, small yellow specks, barely visible to the unaided eye, will be noticed. These little specks are portions of the actinomyces, which cause the disease. Sometimes the pockets of pus are so filled with these minute organisms that they present a crumbling appearance. Usually these pus cavities are connected with each other by small sinuses, though not always. These small pockets may be separated from each other by bands of fibrous tissue which are distributed through the substance of the tumor.

If the tumor is caused by a bulging of the bones of the head, as is the case whenever the organism gains entrance and commences growing in the interior of the bone, the bone tissue in the interior becomes disintegrated and absorbed in places, and pockets are formed containing nests of actinomyces, as in muscular tissue. While the interior of the bone is being broken down by the action of this disease, the diameter of the bone is increased by the deposition of new material, until it may be several times its normal size, and the interior be completely honeycombed as a result of this disease.

This bulging and honeycombed condition of the bones is illustrated in plate III. Figure 1 is the jaw of the animal shown in plate I. The surrounding tissue has been removed to show the increased size of the jaw bone. The darker spots on either side of the molar teeth are openings through which pus was discharged into the mouth. These openings are nearly filled with a growth of neoplastic tissue from the interior of the tumor. Figure 2, same

PLATE III.

Jaw-bone, showing effects of actinomycosis.

FIG. 1. FIG. 2.

plate, was intended to show the honeycombed condition of the bone, but is not very satisfactory, it being difficult to show by means of a photograph.

The tumor shown in figure 1 was about eight months' development; figure 2 about five years.

HISTOLOGICAL EXAMINATION OF TUMOR.

Examined microscopically, the muscular tissue in the immediate vicinity of the tumor seems to be undergoing a gradual disintegration. Many of the fibres are much smaller in diameter than normal, the

Actinomyces, magnified 500 diameters.

striæ are less distinct, and the fibres are paler than usual. Occasionally muscular fibres are found which are enlarged several times their normal diameter and filled with a granular protoplasm.* In these fibres in some cases I have found what appears to be the mycelium of the actinomyces running lengthwise through the fibre, and at some point in the muscular fibre there seems to be a rosette forming. Between the muscular fibres, and throughout the substance of the tumor large numbers of cell nuclei are found, which stain deeply. In bone tissue that is slightly affected, large numbers of osteophytes are found, but in case the bone is very badly diseased the bone cells seem to be few or wanting entirely.

### CAUSE OF THE DISEASE.

This disease is due to the growth in the animal tissues of a peculiar vegetable organism, named from the radiating or star-shaped structure "actinomyces." The rosette or radiating portions of this fungus are very numerous in the pus from an actinomycotic tumor, and appear to the unaided eye as minute specks. These little specks are collections (rarely single) of rosettes. A single rosette is shown in figure 1, plate IV, as it appears when flattened slightly and examined under a compound microscope. The rosettes vary much in size, not only in different animals, but in the same animal, ranging from 10mm to 200mm, 30 to 40mm being the prevailing size. The largest rosettes observed were probably not single, though it was difficult to determine, as they seemed to coalesce.

The rosettes are composed of a number of club-shaped structures which radiate from the centre of the mass. These club-shaped bodies vary as much in size as do the rosettes. Figure 2, plate IV, shows this variation in size. From 1 to 10mm are common measurements, though occasionally some are found which exceed these. Figures 3, 4, 5, 6 and 7 show different shaped clubs that occur. The club-shaped bodies do not reach to the centre of the rosette, but are connected with it by a fine thread-like structure, which is shown in figures 8 and 9. This thread-like structure is not readily demonstrated, for, in tearing out or crushing the rosette, the clubs break off at their junction with this thread. Some investigators have mentioned a polymorphous form of actinomyces in which coccoid and rod-shaped structures are found. The only

---

* Dr. Heneage Gibbes considers these granules of protoplasm as rays of the actinomyces, bu to me this granular mass seems to be altered protoplasm of the muscular fibres, caused by the growth of the mycelium. I have been unable to demonstrate a connection between the mycelium and the granules of protoplasm.

coccoid appearance which I have observed is in focusing on a rosette, the ends of the clubs first appear, as shown in figure 10; but that these coccoid bodies are ends of clubs seems too evident to mislead. This polymorphous form will be discussed somewhat under the head of "Culture Experiments.'

Figure 11 shows what may be called a monstrosity, and one of such size and shape is rarely met with. I am unable to determine whether it is an exaggerated club-shaped portion or a portion of the mycelium; probably it belongs with the club-shaped portions.

If a piece of neoplastic tissue which forms in an actinomycotic

PLATE V.

PLATE VI.

Photo-micrographs of actinamyces in tissues.

tumor, and which usually contains the actinomyces in an active, growing stage, is allowed to decompose in a wet chamber, and the detritis carefully washed away with distilled water, structures such as are shown in Nos. 12, 13, 14, 15, 16 and 17 may be found. It will be noticed that these portions of the organism are much longer and not as thick as the clubs which form the rosettes. These slender threads are the mycelia, or growing portions of the organism which penetrate new tissue and thus extend the sease.

The mycelial threads, shown in figures 13, 14 and 15, are wider in some portions than in others. In the narrowest places the walls seem to touch each other. Whether this irregularity is natural or the result of twisting the mycelial thread I am unable to determine. I have not been able to demonstrate partitions in the mycelium, the apparent partition in figure 14 being a twist of the mycelium. This widening and narrowing of the mycelium is so constant as to lead one to the opinion that the mycelial thread is greater in one diameter than the other, and the irregular outline may be due to twisting.

In figures 15 and 16 the mycelia were pushing out from a rosette. The mycelia are much more difficult to stain than the clubs of the rosettes. In figures 16 and 17 the mycelia seem to branch, but a careful manipulation of the specimens did not demonstrate this, and I have failed to find specimens that were certainly branched.

The mycelia are rarely found in rosettes, as they occur in the pus from the tumor. The rosettes in the pus are larger, the club-shaped bodies are much thicker, and stain more readily than the rosettes found growing in the tissues.

MANNER OF GROWTH IN TISSUES.

It is extremely difficult to trace the growth of the actinomyces through the tissues. The mycelia are so small, and as they do not run any distance in the same plane, it is impossible to get more than a very small portion in the focus of a lens of a sufficient power to show the mycelium. I have found it impossible to stain the mycelia, but by staining the surrounding tissues, the mycelia, on account of the difference in the refraction of light, appear as very bright threads. For staining sections, I have had the best results from a double stain, picro-carmine and Spiller's purple, recommended by Doctor Gibbes. Muscular tissue I have found the best for demonstrating the mycelia, as there seems to be a tendency of the mycelium to follow the course of a muscular fibre, but even here it is impossible to follow it but a little distance. The mycelium is more readily seen close to the free extremity on account of its greater size. Neoplastic tissue ranks next to muscular tissue in ease of demonstrating the mycelia. They can be found in other affected tissues, but not as readily, except in white fibrous connective tissue, where I have been unable to demonstrate the mycelium to a certainty. While the mycelial threads are undoubtedly present, the highest refractive power of the fibrous connective tissue makes the demonstration of the mycelia extremely difficult, if not impossible. The mycelial threads can be demonstrated in almost all the affected tissues, but I have been unable to find them in adjacent tissues that were apparently healthy.

At varying distances along the mycelia rosettes are formed, and can be readily seen in sections. Occasionally they appear as single rosettes, as in plate V, but the most common form is a cluster of rosettes which coalesce and form a nest, as is shown in plate VI. The photo-micrographs, from which these cuts were made, were taken with a Zeiss 2.5mm. objective from sections of neoplastic tissue, and are magnified 400 diameters. I found it impossible to make satisfactory drawings, and the photo-micrographs only show the general structure, as it is impossible to get the organism in a single focal plane.

### CULTURE EXPERIMENTS.

Attempts were made to grow the actinomyces in various culture media outside the animal economy, in order to study the various stages in the life history of the organism, and to furnish, if possible, material for inoculation. Over 300 trials were made under the following conditions: 120 were made upon agar agar, plain and nutrient, 58 upon nutrient gelatine, 60 upon blood serum, 40 in bouillon, and others upon sterilized egg and potato. They were tried at the temperature of the room, and in an incubator at a temperature of 100 degrees F. Surface and anærobic cultures in gelatine and agar agar, and surface cultures on blood serum in an atmosphere of hydrogen were tried, but all were unsuccessful, and in no case was I able to get a marked growth of actinomyces.

The greatest difficulty encountered was to obtain the actinomyces free from bacteria, and as soon as the actinomyces were placed in culture media the bacteria developed very rapidly. In spite of precautions taken in collecting and thorough washing with distilled water, less than 10 per cent. were free from bacteria. The bacteria occurring in cultures were those commonly found in pus; staphylococcus pyogenes albus and aureus, together with micrococci and bacilli. Inoculation with pure cultures of the bacteria produced no serious results. It sometimes occurred that, in a flask inoculated with actinomyces, the micrococci would develop rapidly, and, in the course of ten days, would be superseded by bacilli. To a superficial observer it might appear that the actinomyces changed into micrococci and then into bacilli, and these circumstances may have given rise to the "polymorphous" form of actinomyces, mentioned by some investigators. In these cultures I have always found the actinomyces in their original form, and cultivation of the bacteria through successive generations invariably gave pure cultures of the original micrococcus or bacillus. This is the nearest to a "polymorphous form" of the actinomyces that I have observed.

In studying the action of actinomyces upon an artificial media I found that when kept upon agar agar for nearly four months they made no growth that was apparent on a careful examination, though the organism appeared as fresh as when first put in. In some cases the clubs appeared slightly swollen, but careful measurements and comparison with fresh specimens gave no positive results. Tufts or rosettes of actinomyces were measured and their general appearance noted as closely as could be, and then placed in culture tubes or flasks, and after variable periods compared with the original measurements, but no positive indications of growth were noted. In a few cases mycelial threads seemed to have pushed out a short distance from rosettes that were obtained from fresh neoplastic tissue, but in any case they were no longer than those found in rosettes taken directly from the tissue. Short mycelial threads can be found proceeding from rosettes taken from tissue where the actinomyces are growing rapidly, but are rarely found in rosettes as they occur in pus discharged from a tumor.

The actinomyces show great resistance to decomposition. If a piece of tissue containing the organisms is allowed to decompose in a wet chamber, the actinomyces retain their original appearance. If pus containing the rosettes is allowed to dry upon glass, by soaking in water a short time they regain their original appearance. I have kept material in this manner for two years in the laboratory, and after soaking a short time the rosettes look as fresh as when first obtained. The actinomyces also show great resistance to stains. Gram's method, Bismarck brown and gentian violet, gives fair results. Spiller's purple has given the best results. The actinomyces can be studied very nicely without staining, by using a high power and changing the light. Specimens can be preserved nicely by mounting in glycerine.

### INOCULATION EXPERIMENTS.

An attempt was made to inoculate animals and produce the disease by using the pus which escaped from a tumor, and which contained large numbers of the rosettes. In the inoculations made the pus was examined microscopically, to be certain that it contained the organisms.

The animals used were guinea pigs, one dog, two two-year-old steers, and two heifers—one three-year-old, which was inoculated only once (No. 3), and one yearling heifer, a small Jersey.

All the material used for inoculation was taken directly from a tumor and transferred to point of inoculation, except in Nos. 1 and 2. This pus had been kept nearly forty-eight hours, and was somewhat decomposed. In Nos. 3, 4, 5 and 6, the pus was kept about twenty-four hours.

INOCULATIONS.

| *No.* | *Kind of Animal.* | *Region.* | *Material used.* | *Results.* |
|---|---|---|---|---|
| 1.. | Guinea-pig | Shoulder | Pus | Œdœma; pig sick for two days; healed. |
| 2.. | " | Hip | " | Œdœma; pig sick for three days; died of septæmia. |
| 3.. | Heifer, 3 years old | Neck | " | Abscess formed and discharged; healed in 10 days. |
| 4.. | Dog | Shoulder | " | Healed. |
| 5.. | Guinea-pig | Neck | " | " |
| 6 | " | Shoulder | " | " |
| 7.. | Steer, 2 years old | " | " | " |
| 8.. | " " | " | " | " |
| 9.. | " " | Neck | " | " |
| 10.. | " " | Side of jaw | " | " |
| 11.. | " " | Shoulder | " | " |
| 12.. | " " | " | " | " |
| 13.. | Guinea-pig | Hip | " | " |
| 14.. | " | Shoulder | Neoplasm | Healed for 10 days, then grew and formed tumor. |
| 15 | " | " | " | Gave signs of growing, but finally healed. |
| 16.. | Heifer, 1 year old | " | Pus | Healed. |
| 17.. | " " | Neck | Neoplasm | Grew. |
| 18.. | Steer | Back | " | Gave promise of growing, but did not. |
| 19.. | Heifer | Shoulder | Pus | An abscess was formed artificially and inoculated; healed. |
| 20.. | Steer | Neck | Neoplasm | Healed. |
| 21.. | " | Shoulder | " | Grew. |
| 22.. | Guinea-pig | Hip | Pus | Healed. |
| 23.. | Steer | Tongue | " | " |
| 24.. | " | Shoulder | " | " |
| 25.. | " | Side of jaw | " | " |
| 26.. | Heifer | Shoulder | Neoplasm | Grew. |
| 27.. | " | Neck | " | " |
| 28.. | " | Jaw | " | Gave signs of growing, but finally healed. |
| 29.. | " | Thigh | Pus | Healed. |
| 30.. | Steer | Neck | " | About one ounce of pus was used; an abscess formed, discharged and healed. |
| 31.. | Guinea-pig | Back | " | Healed. |
| 32.. | Heifer | Shoulder | Neoplasm | Grew. |
| 33. | Steer | " | " | Healed. |
| 34.. | " | Jaw | Pus | " |
| 35.. | Heifer | Parotid gland | " | Considerable Œdœma; hard bunch formed, but disappeared in three weeks. |
| 36.. | " | Jaw | Neoplasm | Grew. |
| 37.. | Steer | Back | Pus | Healed. |
| 38. | " | " | " | " |
| 39.. | " | Flank | " | " |
| 40.. | " | Shoulder | " | Abscess formed artificially and inoculated; healed. |
| 41.. | Guinea-pig | Hip | " | Healed. |
| 42.. | " | " | Neoplasm | Pig died of septæmia. |
| 43.. | Steer | Neck | Pus | Healed. |
| 44.. | " | Jaw | " | " |
| 45.. | Heifer | Submaxillary | " | " |
| 46.. | " | Inside of thigh | " | " |
| 47 | Steer | Flank | " | " |
| 48.. | " | Submaxillary | " | " |
| 49.. | " | Shoulder | Neoplasm | Grew. |
| 50.. | " | Tongue | Pus | Healed. |
| 51.. | " | Jaw | " | " |

In the 51 inoculations, 37 were made with pus from an actinomycotic tumor which contained rosettes, and none grew to form an actinomycotic tumor. In Nos. 3 and 29 an abscess formed, which, on breaking, discharged pus which contained the rosettes but only those that were placed there with the pus; none grew in the tissues. In Nos. 15 and 34, which gave evidence of growing, a bunch formed such as precedes an abscess, but was finally absorbed. In all four cases a very large quantity of pus was used in inoculation. In Nos. 19 and 39 an abscess was first formed artificially, and pus containing rosettes placed in the cavity of the abscess; both failed. Of the 14 inoculations made with neoplastic tissue, which contain the actinomyces in a growing stage, eight were successful, and a characteristic actinomycotic tumor resulted. Five failed to grow and, in one case, septæmia followed, which destroyed the animal.

In those cases where the disease was produced, the term "transplanting" will express the conditions more clearly, because tissue which contained the growing organism was transferred to another animal, or another part of the same animal, and thus produced the disease.

In all cases where the inoculations were successful, the wound healed rapidly and only a small fibrous bunch remained. In the course of from 14 to 27 days this began to enlarge, and assumed the characteristic appearance of this disease.

One case of accidental infection occurred in the animals under observation. In the animal illustrated in Plate II, the tumor discharged pus into the mouth through three openings which were nearly filled with neoplastic tissue which protruded into the mouth cavity. This steer contracted the disease in the left lower jaw, between the third and fourth molars. This was not observed until an autopsy was held. Whether infection occurred from pus or from infected feed or from a piece of neoplastic tissue, I cannot say to a certainty. From the results obtained from inoculation, the infection was probably produced by a piece of detached neoplasm. The autopsy also revealed an abscess of the rumen, which was caused by wire nails which the animal had swallowed, penetrating the walls of the rumen. The abscess contained about six ounces of laudable pus, but no actinomyces were present.

Some writers have assumed that the small fibrous tumors, situated along the small intestines and filled with a cheesy pus, which have been observed in some animals, were actinomycotic in nature. These small fibrous tumors are quite as common in cattle not affected with actinomycosis, and the tumors themselves are not actinomycotic in nature.

### NATURE OF THE ACTINOMYCES.

As actinomyces, in their growth in the animal tissues, form mycelial threads with rosettes of club-shaped bodies, they may be classified as a degenerate form of some species of the Ascomycetes, a group of plants which include many of our common fungi. It is generally conceded that the animal tissue is not the natural *habitat* of the actinomyces. They probably grow, naturally, upon other plants, especially upon the *graminæ,* and mature their spores. These spores, when taken into the animal economy with the food, may gain entrance to the tissues through a wound, vegetate, and produce the disease known as actinomycosis. It is probable that the conditions for growth of the organism in the animal tissues are not sufficiently favorable to allow the plant to mature spores; hence the club-shaped bodies are not capable of vegetating.

I have examined many common grasses and grains for antinomyces, but have been unable to find them, though some investigators report success in this direction. I am of the opinion that the actinomyces are so changed in the animal tissue by the different surroundings and conditions for growth, as to be unrecognized. I have tried injecting spores of a number of our common fungi into the animal tissues, in hopes of possibly stumbling upon the fungus that produced the actinomyces, but without success.

### HOW ANIMALS CONTRACT THE DISEASE.

There is a theory that one animal will contract this disease from another by eating food upon which has fallen the pus from an actinomycotic tumor; but experiments show that the disease cannot be transmitted by the rosettes which are found in the pus. Cases can be cited where several animals have contracted the disease in succession as going to prove that one animal contracted the disease from another. In such cases the animal must have contracted the disease from the same source, infected food. On the other hand, cases can be cited where an animal suffering from this disease has mingled freely with many others for a number of years, and no other cases of the disease occurred. Cases also occur upon farms where the disease has never been known before.

The probable mode of infection is by animals eating food upon which the organism which produces the actinomyces is growing; a spore, or possibly a piece of the growing organism, gains entrance into the animal tissues, either through an abrasion of the tissues or opening of a gland, vegetates, and produces the disease known as actinomycosis. In some cases animals undoubtedly contract the disease by inhaling the spores, which may lodge in the sinuses of the head and produce the disease in this region. Animals may become affected with this disease in any region to which the spores of the original fungus may gain access from the outside of the body or through the digestive or respiratory systems, but not through the circulatory system. I have tried to produce this disease by giving animals food mixed with pus from an actinomycotic tumor, but was not successful.

### TREATMENT.

If the tumor is favorably situated, and is treated early and thoroughly, a complete cure may be expected; but if the disease originates within the bones, it usually secures a good foothold before treatment is begun, and in many cases treatment is very unsatisfactory.

There are two general methods of treatment: First, by removing the tumor; second, the iodide of potash treatment. The best and most satisfactory treatment, where it can be applied, is complete removal of the tumor, either with the knife or by using caustic medicines. Of these, the knife is preferable for small tumors. The whole tumor should be removed and the wound treated with some good antiseptic solution, such as corrosive sublimate one part, to 1,000 parts of water. If care is exercised to remove all the diseased tissue, a complete cure may be expected.

Another method of removing the diseased tissue is by the use of caustics. Arsenic or corrosive sublimate is commonly used, a small quantity being wrapped in tissue paper and pushed into the center of the tumor; sometimes, if the tumor is a large one, several pellets of the caustic are pushed into the different parts of the tumor. In the course of from 12 to 15 days the diseased tissue surrounding the caustic sloughs out and the wound is then treated with an antiseptic solution as before. It is often quite difficult to remove all the diseased tissue by the use of caustics, and the tumor may continue to grow. In using caustics it must be remembered that these caustics are irritant poisons, and should not be left on the surface of the tumor, if it is situated so an animal can lick the affected part.

The iodide of potash treatment consists in giving the iodide of potash internally, in from one to three drachm doses, according to the size and age of the animal. The iodide of potash should be dissolved in a pint of water and given as a drench. In the course of a week a condition known as iodism will be produced, there will be a slight discharge from the eyes and nostrils and the epidermis scales off, especially in the region of the neck. The use of the iodide of potash seems to destroy the actinomyces, and the tumor may be absorbed. It is necessary to continue the medicine for two or three months, and the treatment requires much time and is expensive. This treatment has not given good results in my hands For the first two months the tumor is usually absorbed quite rapidly, but it usually reaches a stage where further treatment is useless. I have found the hypodermic injection of a weak solution of iodine (.05 per cent.) in the affected tissues aids materially in the absorption of the tumor.

### IS THE FLESH DANGEROUS AS FOOD?

As this disease is purely local in character, and does not extend beyond the tissues visibly diseased, there is no danger of contracting the disease from eating the flesh of affected animals, provided the diseased portions are removed. While a few cases of actinomycosis in man have been reported in this country (less than a dozen), there is no evidence whatever that they contracted the disease from the flesh of affected animals. When this disease occurs in man it must be considered as originating from the same source as in cattle—that is, from infected grasses or grains. I do not wish to be considered as advocating that all animals suffering from this disease should be slaughtered for food. Whether the flesh of actinomycotic animals is a *proper* article of food must depend upon circumstances. If the animals are in good condition and the tumor is small, I should consider the flesh of such an animal as suitable for food; but if the animal is thin, or the tumor large or discharging pus freely, they should be condemned, not because the flesh is dangerous as food, but because it is not a proper or suitable food. To illustrate: If an apple, otherwise perfect, contains a small decayed spot, if the decayed portion is removed I should consider the remainder suitable for food. If the apple is small and the decayed portion extensive, it should be rejected, not because it is dangerous, but because it is not suitable for food.

### CONCLUSIONS.

*Actinomycosis bovis*, or lump jaw of cattle, is a parasitic disease caused by the growth in the tissues of a fungus called actinomyces.

It appears as a lump or tumor usually in the region of the head or neck, and may grow to a large size. This tumor usually discharges a yellowish pus, which contains portions of fungus known as actinomyces. It is not transmissible from one animal to another by means of the actinomyces as they are found in the pus. It can be transmitted to other cattle by inoculating with a piece of tissue from the tumor which contains the organism in a growing state. The actinomyces, which cause this disease, are probably a degenerate form of some fungus which grows naturally upon feed stuffs or grain. When the spores of the original fungus are taken into the animal economy, they may gain entrance to the tissues, vegetate, and produce the disease known as *Actinomycosis bovis*, or lump jaw. There is no danger of persons contracting this disease from eating the flesh of affected animals, provided the visibly diseased portion is removed.

The treatment consists in removing the tumor, either with a knife or by the use of caustics. The iodide of potash given internally may effect a cure.

## Figures and Tables

**PLATE I. f1:**
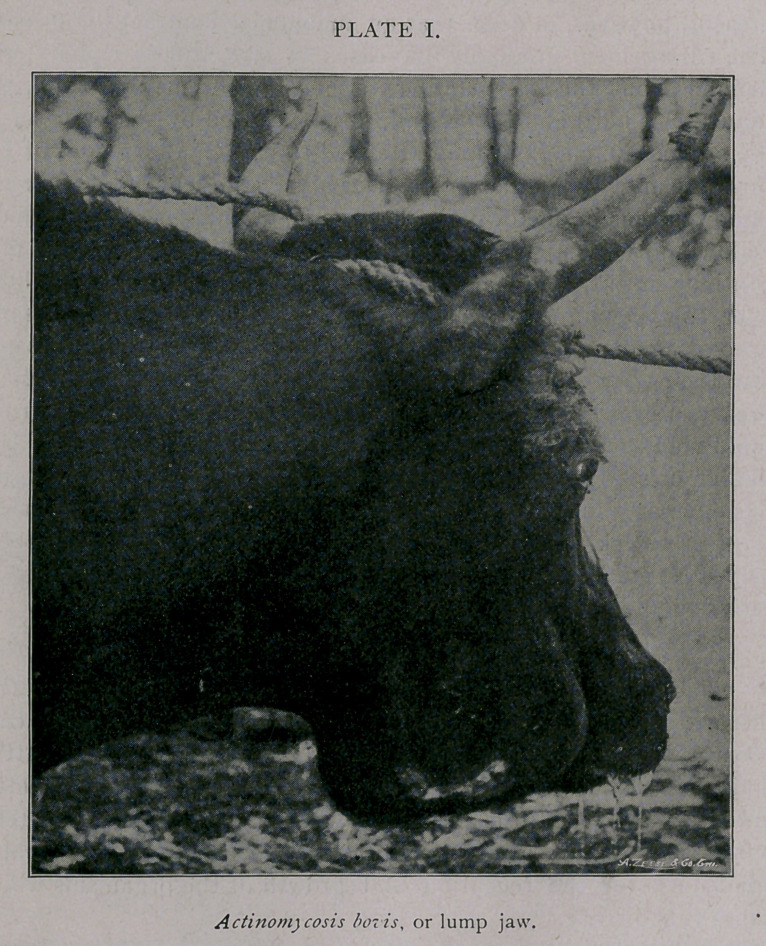


**PLATE II. f2:**
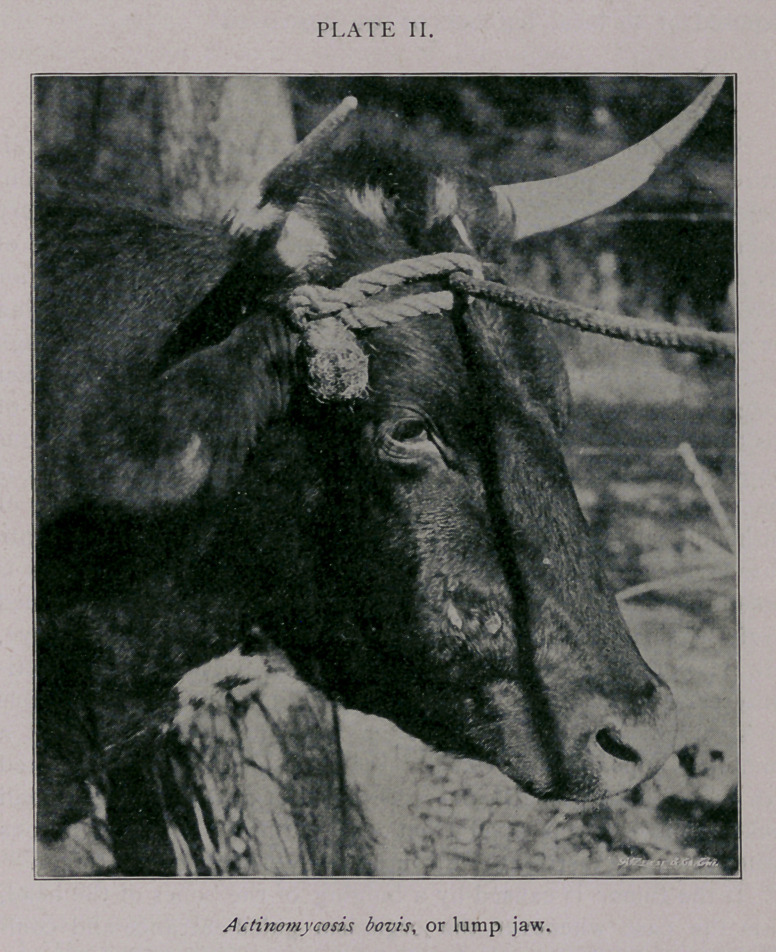


**PLATE III. f3:**
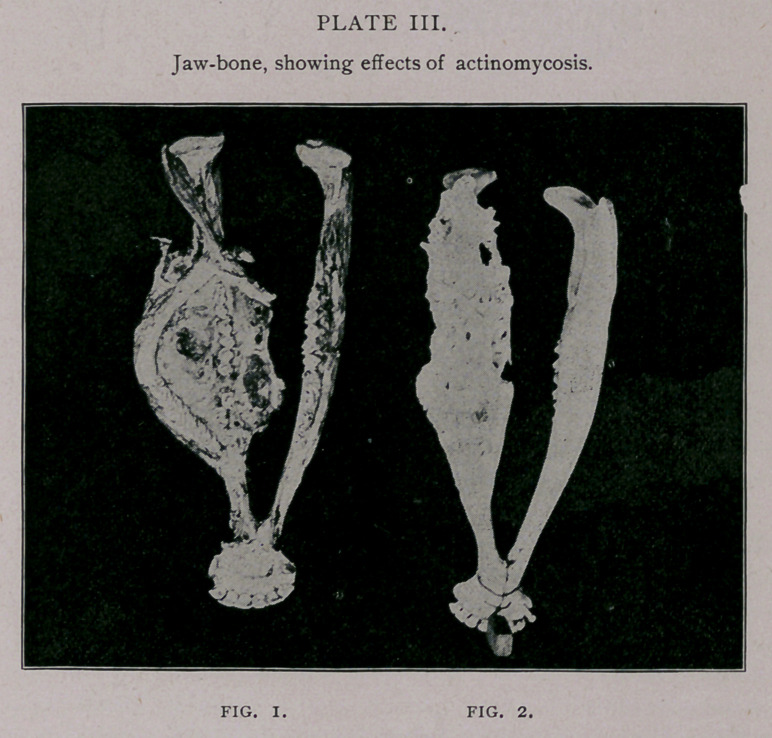


**PLATE IV. f4:**
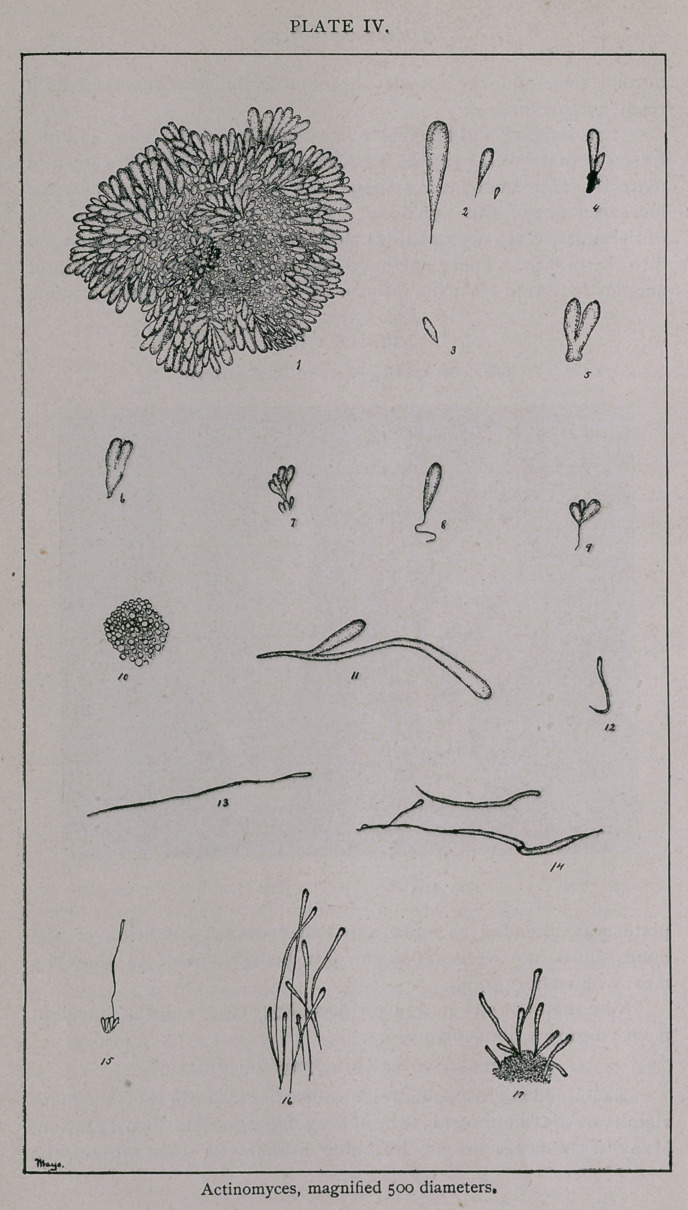


**PLATE V. PLATE VI. f5:**